# Platelet-Derived Soluble CD40L and Its Impact on Immune Modulation and Anti-IL6R Antibody Treatment Outcome in Rheumatoid Arthritis

**DOI:** 10.3390/cells14090625

**Published:** 2025-04-22

**Authors:** Carlos Zamora, Cesar Diaz-Torne, Maria Angels Ortiz, Patricia Moya, Hye Sang Park, Concepció Pitarch, Elisabet Cantó, Ruben Osuna-Gomez, Maria Mulet, Maisa Garcia-Arguinzonis, Diego Collado, Hector Corominas, Silvia Vidal

**Affiliations:** 1 Inflammatory Diseases, Institut Recerca Hospital Sant Pau (IIB Sant Pau), 08041 Barcelona, Spain; carlosza86@gmail.com (C.Z.); mortiz@santpau.cat (M.A.O.); ecanto@santpau.cat (E.C.); rosuna@santpau.cat (R.O.-G.); mmulet@santpau.cat (M.M.); 2 Rheumatology Department, Hospital Santa Creu I Sant Pau, 08041 Barcelona, Spain; cesardiaztorne@gmail.com (C.D.-T.); pmoyaa@santpau.cat (P.M.); hsang@santpau.cat (H.S.P.); cpitarchg@csi.cat (C.P.); hcorominas@santpau.cat (H.C.); 3 Institut Recerca Hospital Sant Pau (IIB Sant Pau), 08041 Barcelona, Spain; mgarciaar@santpau.cat; 4 Centre Medic Teknon, 08022 Barcelona, Spain; medilubo@gmail.com; 5 Department of Medicine, Universitat Autonoma de Barcelona, 08193 Barcelona, Spain

**Keywords:** platelet immunomodulation, rheumatoid arthritis, T lymphocyte activation, CD40L, cytokine production

## Abstract

Background: Platelets (PLTs) from healthy donors (HD) modulate T lymphocyte responses but PLTs from rheumatoid arthritis (RA) patients contribute to persistent systemic inflammation. This suggests that PLTs from RA patients and HD have different immunomodulatory effects. Methods: Using cell culture, flow cytometry, proteomics, and ELISA, we compared PLTs from HD and RA patients and their effects on T lymphocyte activation and cytokine production. Results: HD PLTs suppressed T lymphocyte proliferation and IFNγ and TNF production, while RA PLTs exhibited reduced suppressive capacity. In the presence of RA PLTs, IFNγ levels correlated with T lymphocyte proliferation, greater disease activity, and anti-citrullinated protein antibodies (ACPA). Proteomic analysis revealed that RA PLTs show upregulation of proteins linked to acute-phase response and complement activation. RA PLTs secreted higher levels of soluble CD40L (sCD40L) and PDGF-BB that correlated with enhanced IFNγ production. Seropositive RA patients had higher levels of sCD40L, and these levels were predictive of disease remission in RA patients treated with anti-IL6R. sCD40L was found to enhance T lymphocyte activation and to contribute to increased pro-inflammatory cytokine production. Conclusions: This study highlights the diminished ability of RA PLTs to suppress T lymphocyte activation and that sCD40L can be a potential biomarker and therapeutic target in RA.

## 1. Introduction

Rheumatoid arthritis (RA) is a chronic autoimmune disease characterized by persistent synovial inflammation and joint destruction, leading to significant morbidity and disability. The immune dysregulation in RA involves complex interactions between immune cells, autoantibodies, and inflammatory mediators that drive both local and systemic inflammation. While T lymphocytes and macrophages are key contributors to the inflammatory milieu in RA [1,2], an emerging body of evidence suggests that PLTs also play a critical role in modulating immune responses in autoimmune diseases, including RA [3]. PLTs seem involved in the cellular activation (immune, endothelial, and stromal cells), synovial inflammation, and chronic inflammation of RA patients.

In addition to their role in clot formation, platelets (PLTs) release a wide range of bioactive molecules that can modulate leukocyte function, such as transforming growth factor (TGF)-β, platelet factor (PF)-4, soluble CD62P (sCD62P), and soluble CD40L (sCD40L). TGF-β is a potent anti-inflammatory cytokine that is able to decrease IFNγ and IL-17 production, T lymphocyte proliferation, the expression of cytolytic molecules, and Treg differentiation [4,5]. PF-4 induces an inflammatory state of monocytes and neutrophils [6], while it inhibits T lymphocyte function but enhances Treg differentiation [7]. sCD62P induces the production of inflammatory cytokines by monocytes and NETosis by neutrophils [8,9]. sCD40L plays a multifaceted role in the immune system. sCD40L supports B cell isotype switching and decreases the production of TNF and IL-6 and increases IL-10 production by monocytes [10,11]. Despite its anti-inflammatory effects in certain contexts, in RA, sCD40L contributes to monocyte activation, leading to the release of proinflammatory cytokines, including TNF and IL-6, which drive joint inflammation and tissue damage. Elevated levels of sCD40L have been observed in subgroups of RA patients [12], correlating with disease activity and the presence of autoantibodies such as anti-cyclic citrullinated peptide antibodies (ACPA) and rheumatoid factor (RF) [13]. In addition to all these PLT-derived soluble factors, a direct interaction of PLTs with leukocytes generates PLT-leukocyte aggregates with immunoregulatory effects [14]. We, along with other authors, have shown that CD4+ T lymphocytes with bound PLTs produce lower levels of IFNγ and IL-17 and proliferate less than CD4+ lymphocytes without bound PLTs [15,16].

The altered function of PLTs in RA garnered interest, particularly regarding their potential to modulate T lymphocyte responses. Previous studies have shown that RA PLTs exhibit a pro-inflammatory phenotype, marked by increased expression of activation markers and the release of cytokines and growth factors [17]. However, the precise mechanisms by which PLTs contribute to immune dysregulation in RA, particularly in the context of T lymphocyte activation, remain poorly understood.

Given these observations and our previous results [16,18,19], we aimed to comprehensively assess the role of PLTs in modulating T lymphocyte responses in RA. In this study, we compared the effects of PLTs from healthy donors (HD) and RA patients on T lymphocyte proliferation, blast formation, and cytokine production. We further examined the concentration of key PLT-derived molecules, and their correlation with immune activation and disease activity. Additionally, we evaluated the potential of sCD40L as a biomarker for predicting treatment response in RA patients undergoing tocilizumab therapy. Our findings provide new insights into the altered regulatory role of PLTs in RA and underscore their potential as therapeutic targets in the management of this chronic autoimmune disease.

## 2. Materials and Methods

### 2.1. Study Subjects

Ten mL of whole blood from healthy donors (HD) (*n* = 40) and patients with active RA (*n* = 92) were collected in BD Vacutainer heparinized tubes (BD Bioscience, Franklin Lakes, NJ, USA). RA diagnosis was based on the 2010 ACR/EULAR Classification Criteria for RA [20]. Table 1 shows the demographics, clinical parameters, and laboratory values of RA patients enrolled in this study. Healthy donors were selected from anonymous blood donors and matched to patients by age and sex. No clinical laboratory data were available for these donors. However, to ensure the absence of inflammatory or autoimmune conditions, all donors completed a general health questionnaire. The study included anonymous consecutive HD and RA patients from Hospital de la Santa Creu i Sant Pau and Hospital Dos de Maig. Patients were treated with an anti-IL-6 receptor (anti-IL-6R) monoclonal antibody, administered according to the standard clinical guidelines. Disease activity was evaluated at baseline and after 24 weeks of anti-IL6R antibody treatment using the disease activity score in 28 joints erythrocyte sedimentation rate (DAS28-ESR) [21]. Clinical remission was defined as DAS28 < 2.6. Written informed consent was obtained from each patient and ethical approval for the study was granted by the Research Ethics Committee of Sant Pau Research Institute (IIBSP-PLA-2021-36, approved 2021).

### 2.2. Isolation of Cells, PLTs, Collagen-Activated PLT Supernatants, and PMPs

Peripheral blood mononuclear cells (PBMCs) were isolated by gradient centrifugation (Lymphoprep, AXIS-SHIELD PoCAs, Oslo, Norway). PBMCs were washed twice with PBS and cryopreserved until use. When needed, cells thawed and adjusted with complete RPMI medium at 1 × 10^6^ cells/mL. PLTs were isolated and frozen using a validated protocol based on Valeri’s method which preserves in vivo recovery, lifespan, and hemostatic function [22]. This allowed uniform experimental conditions across patient and control samples. Briefly, to obtain PLT-rich plasma (PRP), whole blood was centrifuged at 250× *g* for 15 min to separate plasma and PLTs from red blood cells. The resulting PRP supernatant was carefully transferred to cryogenic vials in 6% of DMSO (Merck, Darmstadt, Germany) and kept at −80 °C until use. When needed, frozen PRP was rapidly thawed at 37 °C after 1:1 dilution with complete RPMI medium 5 mM of EDTA (Merck) to avoid PLT activation. To remove debris and contaminating cells, an initial low-speed centrifugation at 150× *g* for 10 min was performed. The resulting supernatant was then subjected to centrifugation at 1500× *g* for 15 min to pellet the platelets. The PLT pellet was washed in calcium-free phosphate-buffered saline (PBS), resuspended and adjusted at 4 × 10^7^ PLTs/mL. The absence of contaminating leukocytes was confirmed by flow cytometry. According to this protocol, the potential residual plasma proteins were significantly diluted (approximately 1000 times).

In those experiments that required the activation of PLTs (to obtain supernatant and PLT microparticles, PMPs), we washed PLT pellets to remove DMSO and EDTA, and the culture was performed in the presence of 1.8 mM CaCl_2_ to restore platelet functionality. PLTs were cultured with 20 µg/mL of collagen type I (Sigma-Aldrich, St Louis, MO, USA) for 90 min at 37 °C and centrifuged at 3000× *g* for 10 min to discard PLTs. Supernatants containing PMPs and soluble factors were then centrifuged at 16,700× *g* for 1 h to pellet PMPs. PMPs were adjusted at 4 × 10^7^ PMPs/mL and supernatants were 0.1 µm filtered to obtain a free PMP collagen-activated supernatant. 

### 2.3. PBMC and PLT Staining for Flow Cytometry Analysis

PBMCs were stained with fixable viability dye (Thermo Fisher Scientific, Waltham, MA, USA), anti-CD3 Viogreen (Miltenyi Biotec, Bergisch Gladbach, Germany), anti-CD4-PECy7 (clone RPA-T4), CD8-PERCP (clone T8) (BioLegend, San Diego, CA, USA), and CD41a-APC (clone HIP8) (Immunotools, Friesoythe, Germany) monoclonal antibodies, and then washed and resuspended with 400 µL of PBS. Live CD4+ and CD8+ T lymphocytes were gated as CD4+ cells or CD8+ cells. Cells with bound PLTs were identified as CD41a+. PLTs (10^6^ in 100 µL) were incubated with anti-CD41a-FITC (clone HIP8) (Immunotools), anti-CD62P-PECy7 (clone AK4), anti-PAC-1-AlexaFluor 647 (clone pac-1) (BioLegend), anti-CD36 PE (clone CB38) (BD Biosciences, Franklin Lakes, NJ, USA), anti-CD63-Vioblue (clone REA563), anti-CD40L-PerCP-Vio700 (clone REA238), and anti-CD147-APC-Vio770 (clone REA282) (Miltenyi Biotec) and acquired by flow cytometry. PLTs and PMPs were identified as CD41a+ events in gate regions previously established using size-calibrated fluorescent beads with sizes ranging from 0.22 to 1.35 µm (Spherotech, Lake Forest, IL, USA). CD41a+ PLT/µL and CD41+ PMP/µL were then quantified. PLT activation was assessed using anti-CD62P-PECy7 mAb. PLT aggregates (PLT aggr) were identified as CD41+ FSC high SSC high. The percentage of CD62P, PAC-1, CD36, CD63, CD40L, and CD147 on PLTs, PLT aggr, and PMPs were obtained according to FMO controls. The percentages of positive cells (% cells) and MFI of each marker were obtained using FlowJo version 10 (FlowJo LLC., Ashland, OR, USA). All samples were acquired using the MACS Quant Analyzer 10 cytometer.

### 2.4. Co-Culture of PLTs with PBMCs

To determine cell proliferation, PBMCs were incubated with 5 µm of 5(6)-carboxyfluorescein diacetate succinimidyl ester (CFSE) (Sigma-Aldrich). CFSE labeled PBMCs were stimulated with an anti-CD3, CD2, and CD28 cell activation/expansion kit (Miltenyi Biotec) for 72 h in the absence or presence of various conditions, including 20:1 PLTs or PMPs per PBMC, collagen-activated supernatants, or 20 µg/mL of collagen (internal control of collagen-activated supernatants). The PLT/PBMC ratio of 20:1 was selected based on our prior studies, where we chose among PLT/PBMC ratios (ranging from 100:1 to 1:1) that reflect the physiological range of PLT and leukocyte counts [23]. This ratio was further validated through coculture experiments and shown to appropriately modulate immune cell responses. Cell proliferation was quantified as CFSE+ cell percentage. Blast lymphocytes were identified as FSC high and gate was established according to unstimulated T lymphocytes.

To assess the effect of sCD40L on PLTs-PBMCs, 0.1–1 µg/mL of sCD40L (Peprotech, London, UK) was added 15 min prior to the co-cultures. Cultured cells were collected and stained for flow cytometry analysis. Culture supernatants were collected and kept at −80 °C until use.

### 2.5. Determination of Cytokine and Chemokine Concentration in Culture Supernatants

IFNγ, TNF (BD Biosciences), IL-17, VEGF, IL-22 (Peprotech), IL-6, sIL-6R, IL-10 (Immunotools), sCD62P (R&D, Minneapolis, MN, USA) PF-4, sCD40L, PDGF-BB (Peprotech), and latent TGFβ (Mabtech, Nacka Strand, Sweden) levels were determined using specific ELISA kits according to manufacturers’ instruction. The limits of detection were as follows: 10 pg/mL for IFNγ, 15.62 pg/mL for IL-17, IL-10, sCD40L, VEGF and IL-22, 20 pg/mL for TNF, 46.87 pg/mL for PF4 and PDGF-BB, 62.5 pg/mL for TGFβ, 125 pg/mL for sCD62P, 4 pg/mL for IL-6, and 50 pg/mL for sIL-6R.

### 2.6. Determination of Cytokine Secretion

Four hours before stopping the culture, PMA (50 ng/mL) and ionomycin (1 μg/mL) (Sigma-Aldrich) were added. After incubation, the stimulated PBMCs were collected and IFNγ, IL-10, IL-17, and TNF secretion were determined using a secretion assay detection kit (Miltenyi Biotec). Additionally, cells were stained with anti-human CD3-Viogreen (Miltenyi Biotec). Samples were acquired with the MACSQuant Analyzer 10 flow cytometer, and the data were analyzed using FlowJo software version 10.

### 2.7. Quantification of Anti-Citrullinated Antibodies and Rheumatoid Factor Levels

ACPAs were determined in plasma using ELIA Unicap (Phadia Laboratory Systems, Uppasala, Sweden) and RF was determined using the Nephelometry system (Beckman Coulter, Miami, FL, USA). Levels of ACPA and RF were expressed as ELIA units/mL and IU/mL, respectively. ACPA levels > 20 ELIA units/mL and RF levels > 26 IU/mL were positive [11].

### 2.8. Proteomics of Platelets

PLTs were isolated from citrated whole blood using the described above centrifugation protocol. After the final wash, PLTs were lysed in buffer containing 1% SDS, protease inhibitors, and phosphatase inhibitors, and protein concentration was measured using a BCA assay. Proteins were reduced with dithiothreitol (DTT), alkylated with iodoacetamide, and digested with trypsin overnight at 37 °C. Peptides were desalted on C18 columns and analyzed by nano-liquid chromatography-tandem mass spectrometry (nano-LC-MS/MS) on an Orbitrap Eclipse (Thermo) at the CRG/UPF Proteomics Unit. Protein identification and label-free quantification were performed using MaxQuant v2.1.4.0 (freely available, downloaded from www.maxquant.org, accessed on 16 April 2025) against the human FASTA file (UP000005640_9606) with a 1% false discovery rate (FDR). Variable modifications included methionine oxidation and N-terminal acetylation, with carbamidomethylation of cysteines as a fixed modification. Minimum razor + unique peptides per protein group was set to 1. Differential expression analysis was conducted using log2-transformed, quantile-normalized LFQ intensities in the Amica platform. Functional enrichment and interaction networks were analyzed with STRING database v11.5 (https://string-db.org/, accessed on 16 April 2025). Proteomic data were generated from a single experiment using PLTs isolated from five HD and five RA patients to compare PLT characteristics in health and disease.

### 2.9. Statistical Analysis

Statistical analyses were performed using Graph Pad Prism version 8 (Graph Pad, San Diego, CA, USA). Data normality was assessed with the Kolmogorov–Smirnov test. Qualitative variables were described as numbers and percentages. A paired *t*-test or Wilcoxon test was used for comparisons between culture conditions, and *t*-test or Mann–Whitney test for comparisons between HD and RA values, depending on data distribution. Correlations were analyzed with Pearson’s or Spearman correlation. A ROC curve determined the optimal cut-off for plasma sCD40L levels to discriminate remission. Data are presented as mean ± SEM, with *p* < 0.05 considered statistically significant.

## 3. Results

### 3.1. Impact of PLTs from HD and RA Patients on T Lymphocyte Stimulation

**Figure 1:**
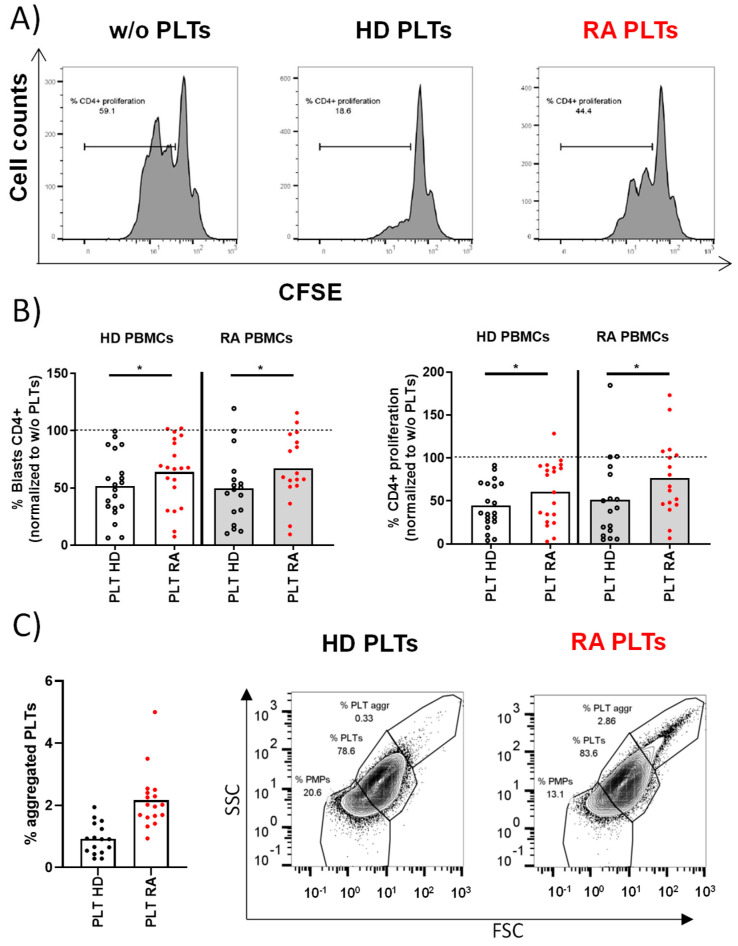
Proliferative response of PBMCs and PLT aggregation in HD and RA patients. (**A**) The proliferative profile of PBMCs from HD activated for 72 h in the presence of HD PLTs, RA PLTs, or in the absence of PLTs (w/o). (**B**) Individual representation of blasts or proliferative PBMCs using HD PBMCs or RA PBMCs activated in the presence of RA PLTs or HD PLTs. (**C**) Proportion of aggregated PLTs in whole blood from HD and RA patients, accompanied by representative dot plots from flow cytometry analysis showing the proportion of PMPs, PLTs, and aggregated PLTs. Statistical analysis was performed using Wilcoxon (**B**) or Mann–Whitney test (**C**), * *p* < 0.05.

T lymphocytes from PBMCs were stimulated in the presence of PLTs from HD or RA patients. After 72 h of co-culture, there were no differences in the percentages of T lymphocytes with bound PLTs regardless of the origin of PBMCs and PLTs (% CD4+ PLT+ in HD PBMCs: 14.84 ± 1.93 for HD PLTs vs. 15.94 ± 2.1 for RA PLTs; RA PBMCs: 14.27 ± 1.93 for HD PLTs vs. 16.72 ± 2.39 for RA PLTs). The proliferation of T lymphocytes was significantly reduced when co-cultured with PLTs from HD (Figure 1A). In contrast, PLTs from RA patients were less effective in suppressing T lymphocyte proliferation (Figure 1B), indicating a disease-specific alteration in PLT function. There was an altered effect of RA PLTs on T lymphocytes regardless of their origin. The comparative phenotypic analysis of PLTs from HD and RA revealed a similar proportion of PLTs and PMPs and a similar expression of CD36, CD63, CD40L, PAC-1, or CD147 (Supplementary Figure S1). However, we found a higher percentage of activated CD62P+PLTs and PLT aggregates in RA patients than in HD (Figure 1C) that may have contributed to the differential impact of RA PLTs on the reduced suppression observed in T lymphocyte proliferation.

### 3.2. Differential Effects of PLTs from HD and RA Patients on Cytokine Production and Correlation with Disease Activity

**Figure 2:**
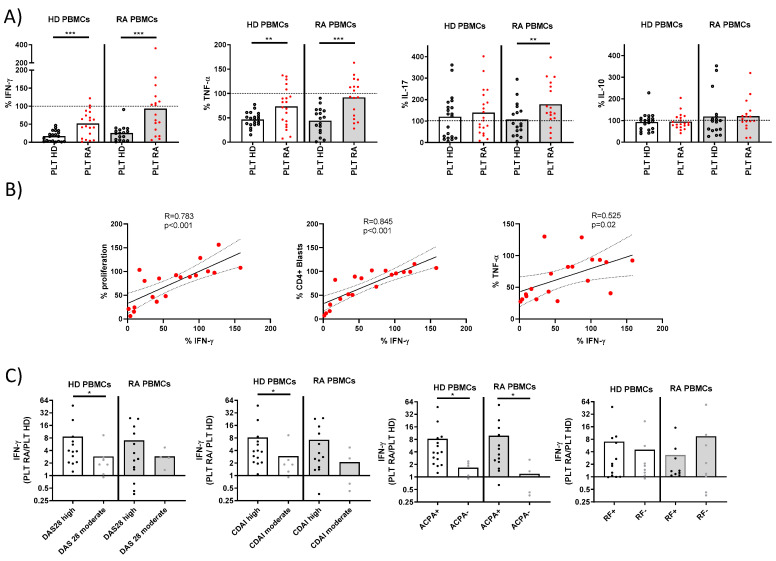
Cytokine production and correlations in PBMC-PLT co-cultures from HD and RA patients. (**A**) Percentage of cells producing IFNγ, TNF, IL-17, and IL-10 was determined in PBMCs from HD and RA patients after activation in the presence of PLTs from HD or RA donors. (**B**) Correlations between proliferation levels, CD4+ blasts, and the percentage of TNF with IFNγ were analyzed in PBMCs from RA patients in the presence of RA PLTs. (**C**) Ratios of IFNγ percentages in PBMC cultures with RA PLTs versus HD PLTs were compared, stratified by the RA disease activity index and the presence of ACPA or RF. Statistical analysis was performed using the Mann–Whitney test (**C**), Wilcoxon test (**A**), and Spearman correlation (**B**). * *p* < 0.05, ** *p* < 0.01, and *** *p* < 0.001.

We next compared the cytokine production by T lymphocytes from PBMCs stimulated in the presence of PLTs from HD or RA patients (Figure 2A). PLTs from HD significantly reduced the production of IFNγ and TNF by PBMCs compared to PLTs from RA patients. This reduction was consistent in PBMCs from both HD and RA patients. Interestingly, neither HD nor RA PLTs had a significant effect on the production of IL-17 and IL-10 by PBMCs from HD. However, a differential effect was observed on IL-17, but not on IL-10, production when PBMCs from RA patients were stimulated, indicating a disease-specific interaction. The IFNγ production in the presence of RA PLTs was strongly correlated with T lymphocyte proliferation, blast formation, and TNF production (Figure 2B). This suggests a coordinated regulation of these immune responses by PLTs, with RA PLTs showing altered effects compared to HD PLTs.

The ratio of IFNγ production in the presence of RA vs. HD PLTs was analyzed for its association with disease activity and serological markers (Figure 2C). A higher IFNγ ratio was associated with increased disease activity, as measured by the Disease Activity Score 28 (DAS28) and the Clinical Disease Activity Index (CDAI), as well as with the presence of ACPA. This finding underscores the potential role of PLT-mediated modulation in IFNγ in the pathogenesis and clinical severity of RA.

### 3.3. Proteomic Profiling of PLTs from HD and RA Patients

**Figure 3:**
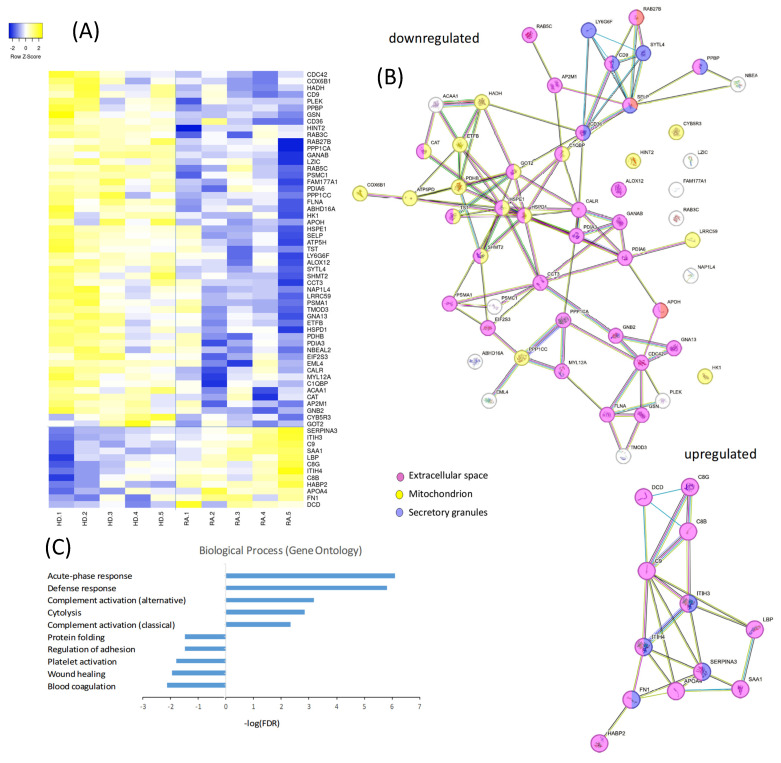
Proteomic and functional analysis of differentially expressed proteins in PLTs from HD and RA patients. (**A**) Heatmap showing the proteomic profiles of PLTs from HD and RA patients. (**B**) Analysis of the compartmental origin of differentially expressed proteins. (**C**) Gene ontology analysis of the top differentially expressed proteins.

A comprehensive proteomic analysis was conducted to compare the protein expression profiles of PLTs from a subgroup of HD and RA patients. The proteomic dataset represents a preliminary comparative analysis based on pooled samples from five RA patients and five healthy donors. The heatmap in Figure 3A illustrates a distinct pattern of protein regulation, with 12 proteins upregulated and 53 downregulated in RA PLTs compared to HD PLTs. The compartmental origin of these differentially expressed proteins was analyzed in Figure 3B. Among the downregulated proteins in RA PLTs, 36 proteins were localized to the extracellular space, 19 to the mitochondria, and 6 to secretory granules. Upregulated proteins were predominantly found in the extracellular space and secretory granules, highlighting specific alterations in PLT function and protein trafficking in RA. Gene ontology (GO) analysis of the differentially expressed proteins revealed significant enrichment in distinct biological processes (Figure 3C). The top five upregulated biological processes in RA PLTs included acute-phase response, defense response, complement activation, and cytolysis. In contrast, the downregulated proteins were primarily associated with blood coagulation, wound healing, PLT activation and adhesion, and protein folding. These findings suggest that RA PLTs are primed for inflammatory responses at the expense of traditional hemostatic and reparative functions.

### 3.4. Differential Effects of Supernatants and PMPs from Activated PLTs on T Lymphocyte Function in RA

**Figure 4:**
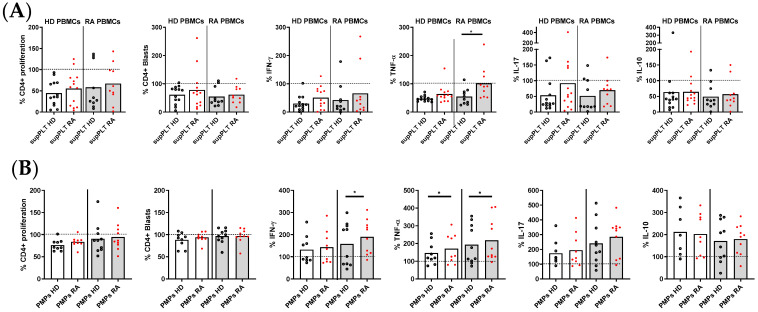
Proliferation and cytokine production in activated PBMCs in the presence of supernatants (sup) or PMPs from PLTs of HD and RA patients. (**A**) Percentage of proliferating and cytokine-producing cells (IFNγ, TNF, IL-17, and IL-10) from activated PBMCs from HD and RA patients in the presence of supernatants from collagen-activated PLTs of HD and RA donors. (**B**) Percentage of proliferating and cytokine-producing cells from activated PBMCs from HD and RA patients in the presence of PMPs from collagen-activated PLTs of HD and RA. Statistical analysis was performed using the Wilcoxon test. * *p* < 0.05.

To decipher whether the impaired function of RA PLTs to regulate T lymphocyte response was due to PLT-secreted factors, supernatants from collagen-activated PLTs of HD and RA patients were analyzed (Figure 4A). The only significant difference observed was that supernatants from RA PLTs exhibited less capacity to inhibit TNF-α production in stimulated T lymphocytes derived from PBMCs of RA patients compared to supernatants from HD PLTs. The impact of PMPs released by activated PLTs from HD and RA patients on T lymphocyte function was also investigated (Figure 4B). Despite the production of collagen-induced PMPs by HD and RA PLTs being comparable, PMPs from RA PLTs were less effective in reducing IFNγ production in T lymphocytes from RA patients, as well as TNF production in T lymphocytes from both RA and HD donors. These findings indicate that soluble factors and PMPs released by RA PLTs have a diminished regulatory capacity on key inflammatory cytokines, potentially contributing to the exacerbated immune responses observed in RA.

### 3.5. PLT-Derived Molecules in Collagen-Activated PLTs and Correlation with IFNγ Production

**Figure 5:**
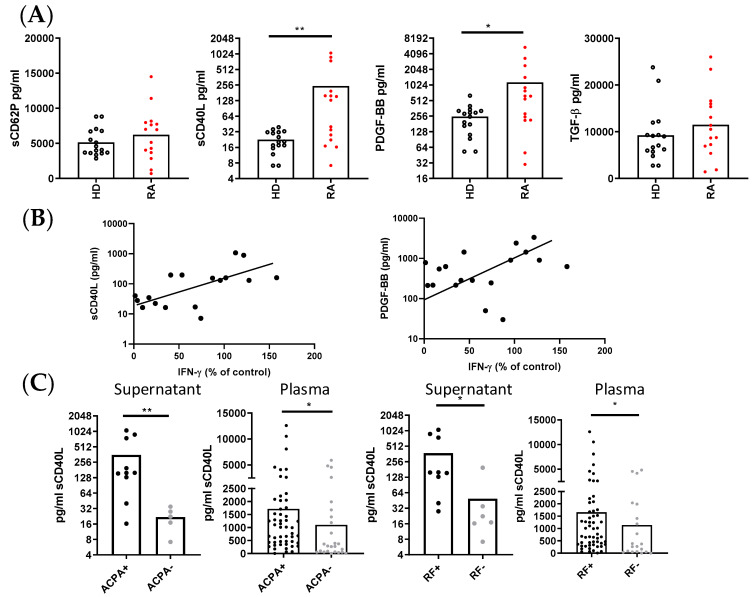
PLT-derived molecules and their immunological correlations in supernatants and plasma from HD and RA patients. (**A**) Concentration of PLT-derived molecules in supernatants of collagen-activated HD PLTs and RA PLTs. (**B**) Correlation between the levels of sCD40L and PDGF-BB in RA PLT supernatants and the percentage of CD4+ T lymphocytes producing IFNγ in PBMCs from HD cultured with RA PLTs. (**C**) Comparison of sCD40L levels in supernatants of activated PBMCs in the presence of PLTs, and plasma from RA patients, stratified by the presence of ACPA and RF. Statistical analysis was performed using the Mann–Whitney test and Spearman correlation. * *p* < 0.05 and ** *p* < 0.01.

We next compared the concentration of several PLT-derived molecules in the supernatants of collagen-activated PLTs from RA patients and HD. The results reveal distinct secretion patterns between the two groups. RA PLTs secreted significantly higher levels of sCD40L and PLT-derived growth factor-BB (PDGF-BB) compared to HD PLTs, while the secretion of sCD62P and TGFβ was comparable between the groups (Figure 5A). In both HD and RA supernatants, there was a correlation between the levels of sCD62P and TGFβ, as well as between the levels of sCD40L and PDGF-BB. Furthermore, the production of IFNγ in co-cultures of RA PLTs and PBMCs showed a positive correlation with the concentrations of sCD40L and PDGF-BB in supernatants of activated PLTs (Figure 5B).

Additionally, when we stratified the RA patients based on their serological status, we observed that seropositive RA patients, defined by the presence of ACPA and RF, had significantly higher concentrations of sCD40L in the supernatants of activated PLTs and in the plasma compared to seronegative RA patients (Figure 5C). This finding underscores the potential link between autoantibody production and PLT activation in RA.

### 3.6. Predictive Value of sCD40L Concentration for Remission in RA Patients Treated with Anti-IL6R

**Figure 6:**
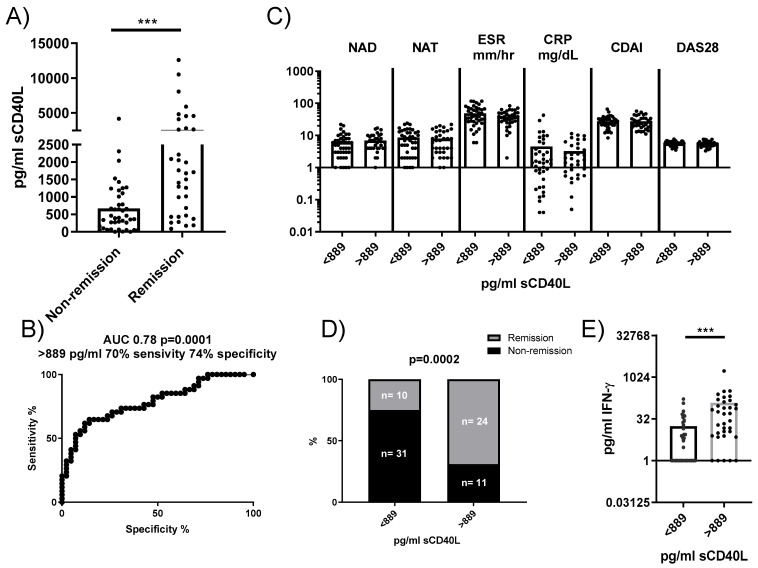
Plasma concentration of sCD40L and its predictive value for remission in patients treated with anti-IL6R antibodies. (**A**) Comparison of plasma concentrations of sCD40L in patients who reached remission versus those who did not after treatment. (**B**) ROC analysis to determine the optimal concentration of sCD40L for predicting remission (cutoff). (**C**) Comparison of baseline characteristics of patients stratified by the sCD40L cutoff. (**D**) Comparison of patient frequencies in the remission and non-remission groups based on this concentration. (**E**) IFNγ concentration in the plasma of patients stratified by sCD40L cutoff. Statistical analysis was performed using the Mann–Whitney test and chi-square. *** *p* < 0.001.

To determine whether our findings could be relevant as predictive biomarkers in RA patients, we analyzed the concentration of sCD40L in plasma and its association with disease remission following tocilizumab treatment. Plasma concentrations of sCD40L were significantly higher in RA patients who achieved remission 24 weeks after anti-IL6R antibody administration compared to those who did not (Figure 6A). A ROC analysis identified a concentration of 889 pg/mL as a threshold, yielding a sensitivity of 70% and a specificity of 74% for predicting remission (Figure 6B). Stratifying patients based on this cutoff showed no significant differences in baseline clinical characteristics between groups (Figure 6C). However, among patients with sCD40L concentrations < 889 pg/mL, only 24.39% patients (10 out of 41) reached remission, whereas 68.57% patients (24 out of 35) with sCD40L > 889 pg/mL achieved remission (Figure 6D). An additional observation was that plasmatic sCD40L levels correlated with plasmatic IFNγ, but not with plasmatic IL-6, sIL-6R, TNF, IL-17, IL-22, and VEGF. Moreover, RA patients with sCD40L > 889 pg/mL had higher levels of IFNγ compared to those with concentrations < 889 pg/mL (Figure 6E), suggesting a potential link between higher sCD40L levels and immune activation pathways.

### 3.7. Impact of sCD40L on PLT-Mediated Regulation of T Lymphocyte Activation

**Figure 7:**
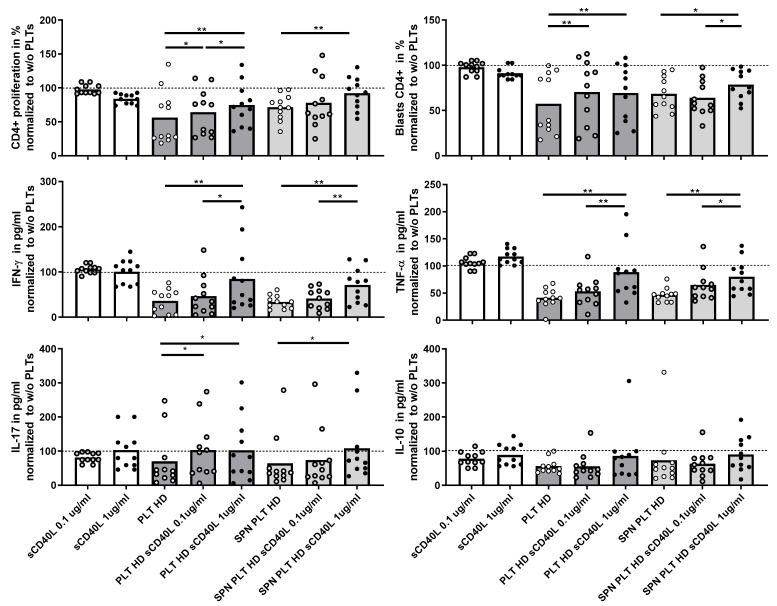
Effect of recombinant CD40L on proliferation and cytokine expression of PBMCs activated in the presence of PLTs. PBMCs were activated in the presence of HD PLTs or supernatant from PLT (SPN PLT) from HD, with two different concentrations of recombinant CD40L (sCD40L) added to the culture for 72 h. The effects of the different concentrations of sCD40L on CD4+ proliferation, blast differentiation, and cytokine expression (IFNγ, TNF, IL-17, and IL-10) were measured and are shown relative to the levels observed in the absence of PLTs. Statistical analysis was performed using the Wilcoxon test for paired samples. * *p* < 0.05 and ** *p* < 0.01.

Given the potential association of sCD40L with immune activation, we investigated whether the presence of sCD40L could affect the regulation of T lymphocyte activation in the co-cultures. Initially, we assessed the direct effect of recombinant sCD40L on T lymphocyte activation by adding two different concentrations of sCD40L to the T lymphocyte cultures. This intervention did not lead to any significant changes in T lymphocyte proliferation, blast generation, or cytokine production, indicating that sCD40L alone does not directly modify T lymphocyte activation (Figure 7). However, when sCD40L was added to co-cultures of PLTs or supernatants of activated PLTs and PBMCs from HD, there was a marked increase in T lymphocyte proliferation, blast formation, and the production of IFNγ, TNF, and IL-17, but not IL-10. These responses reached levels comparable to those observed in cultures without PLTs, suggesting that sCD40L diminishes the ability of PLTs to regulate T lymphocyte activation.

## 4. Discussion

Our study highlights the differential effects of PLTs from RA patients and HD on T lymphocyte activation and immune modulation, emphasizing the altered regulatory role of PLTs in RA and their potential contribution to immune dysregulation and disease pathogenesis.

The co-culture experiments revealed that PLTs from HD significantly suppressed T lymphocyte proliferation and blast formation, confirming previous works that showed the immunoregulatory role of PLTs in maintaining immune homeostasis [16,18,19]. In contrast, PLTs from RA patients were less effective in suppressing T lymphocyte proliferation, indicating a disease-specific alteration in PLT function. Interestingly, this diminished suppression was consistent across T lymphocytes derived from both HD and RA patients, suggesting that the alteration lies in the PLTs themselves, rather than in the T lymphocytes. This impaired regulatory function of RA PLTs may be partly explained by the increased aggregation observed, which may contribute to the reduced immunosuppressive capacity seen in RA. Thus, PLT hyperactivity in RA leads to excessive aggregation, which can alter their immunoregulatory functions by limiting interactions with immune cells and reducing the release of anti-inflammatory mediators such as TGF-β and PD-L1 [24,25]. Additionally, it has been shown that aggregated platelets predominantly release pro-inflammatory factors, further promoting inflammation rather than immune suppression [26]. This aligns with previous reports of heightened PLT activation in RA, suggesting a shift toward pro-inflammatory PLT functions [17,27].

We also demonstrated that PLTs from HD reduced the production of pro-inflammatory cytokines such as IFNγ and TNF in T lymphocytes, whereas RA PLTs had a weaker suppressive effect on these cytokines. This observation was particularly relevant in RA patients, where IFNγ production strongly correlated with T lymphocyte proliferation and TNF production [28,29]. These findings suggest a coordinated regulation of immune responses by PLTs, with RA PLTs demonstrating an altered capacity to modulate cytokine production. Furthermore, the elevated IFNγ production observed in RA patients correlated with disease activity and serological markers such as anti-cyclic citrullinated peptide antibodies and rheumatoid factor, further implicating PLT-mediated immune dysregulation in the severity of RA. This impaired modulation likely contributes to the exacerbated immune activation and systemic inflammation characteristic of RA [30]. Interestingly, RA PLTs specifically altered IL-17 production when interacting with PBMCs from RA patients, pointing to disease-specific interactions between PLTs and T lymphocytes. Our preliminary proteomic analysis revealed significant alterations in the protein profiles of RA PLTs compared to HD PLTs. The observed upregulation of proteins involved in acute-phase responses, complement activation, and cytolysis, alongside the downregulation of proteins associated with hemostasis, demonstrates a functional shift in RA PLTs towards a pro-inflammatory phenotype. This altered protein composition may underpin the reduced capacity of RA PLTs to regulate T lymphocyte activity, as evidenced by their impaired inhibition of pro-inflammatory cytokine production. Supernatants from RA PLTs also exhibited a diminished ability to inhibit TNF production in stimulated T lymphocytes compared to those from HD PLTs. Similarly, PMPs from RA PLTs were less effective in reducing IFNγ and TNF production, indicating a functional impairment in the regulatory properties of both soluble and vesicular components of RA PLTs. These findings provide further evidence that RA PLTs contribute to the heightened inflammatory state observed in RA patients. All these results confirm that RA PLTs are primed for inflammatory responses at the expense of their traditional roles in wound healing and hemostasis [31,32]. While these results provide an initial overview of potentially relevant protein changes, further validation using independent cohorts and complementary techniques is required to confirm the biological significance of the identified differentially expressed proteins.

The increased secretion of sCD40L and PDGF-BB by RA PLTs compared to HD PLTs further supports the notion of PLT-driven immune activation in RA. The positive correlation between the levels of sCD40L and PDGF-BB and IFNγ production in co-cultures of PLTs and PBMCs along with previous publications underscores the role of these PLT-derived molecules in modulating pro-inflammatory responses [12,33,34]. Notably, RA patients with seropositive status, characterized by the presence of ACPA and RF, exhibited significantly higher levels of sCD40L than seronegative patients. This finding suggests a potential link between autoantibody production and PLT activation in RA pathogenesis [13,35]. We also explored the clinical relevance of sCD40L as a predictive biomarker for disease remission in RA patients treated with tocilizumab. Plasma concentrations of sCD40L were significantly higher in patients who achieved remission, and a concentration threshold of 889 pg/mL was identified as predictive for remission with good sensitivity and specificity. Moreover, patients with higher sCD40L levels also had elevated IFNγ levels, further linking sCD40L to immune activation pathways in RA. These results suggest that sCD40L could serve as a valuable biomarker for predicting treatment outcomes in RA. To go even further, we investigated the direct effects of sCD40L on PLT-mediated regulation of T lymphocyte activation. Although recombinant sCD40L alone did not affect T lymphocyte activation, its presence in co-cultures of PLTs and PBMCs from HD enhanced T lymphocyte proliferation and pro-inflammatory cytokine production. These findings suggest that sCD40L interferes with the PLT-mediated suppression of T lymphocyte responses, promoting a pro-inflammatory environment.

In RA, chronic exposure to pro-inflammatory cytokines such as TNF-α, IL-6, and IL-1β induces profound alterations in platelet function at multiple levels, including during their production by megakaryocytes, as well as through metabolic reprogramming, and potential epigenetic modifications. While extensive literature describes the impact of pro-inflammatory molecules on platelet phenotype and secretory profiles, our study specifically focused on analyzing platelets ex vivo from RA patients rather than replicating cytokine exposure in vitro. This approach allowed us to assess how chronic inflammation shapes platelet function in a physiologically relevant context.

Our findings indicate that the immunoregulatory properties of platelets are altered in RA, as evidenced by changes in their proteomic composition and secreted molecules. Despite not detecting significant differences in some surface markers, our data suggest that platelet function is nonetheless compromised, potentially due to intracellular and secretory alterations rather than overt phenotypic changes. This shift likely contributes to the loss of platelet-mediated suppression of T cell activation and instead promotes systemic inflammation, reinforcing the chronic immune dysregulation observed in RA.

While this study provides valuable insights into the role of PLTs in modulating immune responses in RA, several limitations should be acknowledged: RA is a highly heterogeneous disease, with varying clinical phenotypes and treatment responses. A larger cohort with more diverse patient subtypes, including those with different stages of disease progression or varying responses to treatment, would strengthen the conclusions and applicability to broader RA populations. Other factors that influence PLT function, such as concomitant medications, comorbidities, and environmental factors, were not thoroughly accounted for in the study. These variables could have impacted PLT function and immune interactions, potentially introducing confounding effects. While this study primarily focuses on the interactions between PLTs and T lymphocytes, it is important to recognize that the effects of PLTs in RA are unlikely to be limited to lymphocytes alone. Future studies should investigate their interactions with other immune cells, such as monocytes, and neutrophils, to provide a more comprehensive understanding of their immunomodulatory functions in RA. Finally, despite the potential of sCD40L as biomarker for remission, we are aware that relying on a single biomarker may not fully capture the complexity of treatment responses in RA. A multi-biomarker approach incorporating other immune or genetic factors could improve the accuracy and robustness of predicting therapeutic outcomes. Our findings suggest that targeting PLT activation and their derived factors, such as sCD40L and PDGF-BB, could represent novel therapeutic strategies in RA. Inhibition of these pathways might restore the regulatory functions of PLTs, thereby dampening inflammation and improving disease outcomes.

Our findings provide a comprehensive overview of the altered role of PLTs in RA, particularly their impaired capacity to regulate T lymphocyte activation and cytokine production. The identification of sCD40L as a potential biomarker for disease remission and its functional impact on immune regulation underscores the significance of PLT-derived molecules in RA pathogenesis. Targeting PLT-mediated immune responses may represent a novel therapeutic avenue in the management of RA.

## Figures and Tables

**Table 1:** Characteristics of RA patients and healthy donors.

	RA (N = 92)	HD (N = 40)	P
Sex (women) (N)	74	32	ns (#)
ACPA+ (N)	75		
RF+ (N)	60		
Age (years)	60.3 ± 12.9	56.2 ± 12.1	ns (*)
DAS28	5.4 ± 1.2		
SDAI	29.2 ± 11.3		
CDAI	27.4 ± 10.8		
ESR (mm/h)	39.6 ± 22.4		
CRP (mg/dL)	1.7 ± 1.6		
IgG (mg/dL)	1182.1 ± 280		
IgA (mg/dL)	294.1 ± 178		
IgM (mg/dL)	167.3 ± 179		
Monocytes (10^3^/µL)	0.7 ± 0.3		
Neutrophils (10^3^/µL)	6.4 ± 2.9		
Lymphocytes (10^3^/µL)	1.9 ± 0.8		
Platelets (10^3^/µL)	293 ± 80.9		

Comparison by χ^2^ (#) and unpaired Student’s *t*-test (*); abbreviations: ns: not significant SDAI: simple disease activity index; CDAI: clinical disease activity index; ESR: erythrocyte sedimentation rate; and CRP: C-reactive protein.

## Data Availability

Experimental data can be found in tables and figures presented in this manuscript.

## Supplementary Materials

The following supporting information can be downloaded at: https://www.mdpi.com/article/10.3390/cells14090625/s1, Figure S1: Expression and mean fluorescence intensity of surface markers on PLTs, aggregated PLTs, and PMPs.

## Abbreviations

HD	Healthy donors
PLT	Platelets
ACPA	Anti-cyclic citrullinated peptide antibodies
RF	Rheumatoid factor
DAS	Disease activity score
ESR	Erythrocyte sedimentation rate
PBMC	Peripheral mononuclear cell
Aggr	aggregates
PMP	Platelet microparticle
MFI	Mean fluorescence intensity
