# Supplementary material for: Platelet-Derived Soluble CD40L and Its Impact on Immune Modulation and Anti-IL6R Antibody Treatment Outcome in Rheumatoid Arthritis
Source: Cells. 2025 Apr 22;14(9):625. doi: 10.3390/cells14090625 (PMC12071919; doi:10.3390/cells14090625)
Supplement: Supplementary file 1 [file cells-14-00625-s001.zip › cells-3543139-supplementary.pptx]

## Slide 1
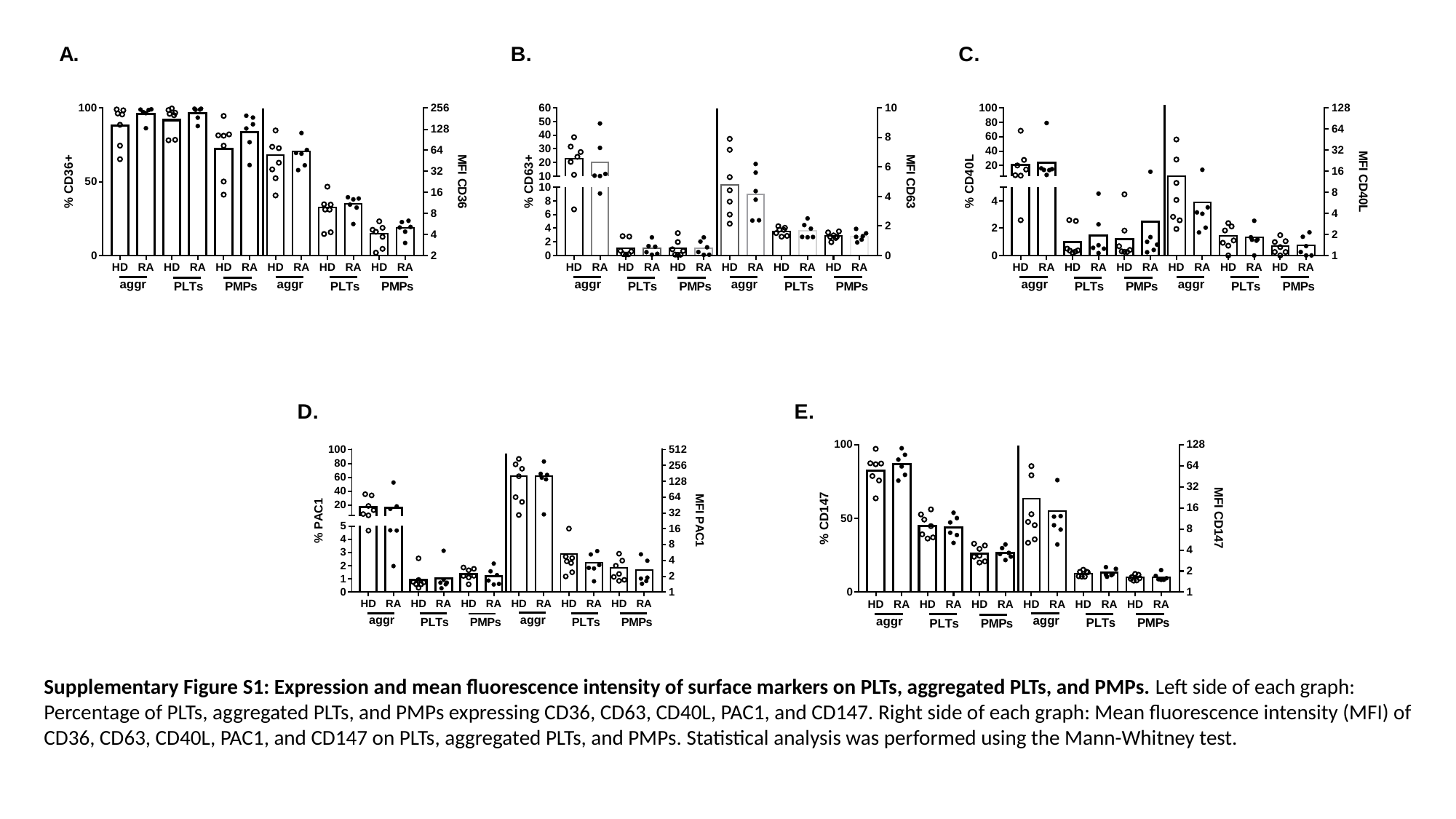

Supplementary Figure S1: Expression and mean fluorescence intensity of surface markers on PLTs, aggregated PLTs, and PMPs. Left side of each graph: Percentage of PLTs, aggregated PLTs, and PMPs expressing CD36, CD63, CD40L, PAC1, and CD147. Right side of each graph: Mean fluorescence intensity (MFI) of CD36, CD63, CD40L, PAC1, and CD147 on PLTs, aggregated PLTs, and PMPs. Statistical analysis was performed using the Mann-Whitney test.
